# Light Management Enhancement for Four-Terminal Perovskite-Silicon Tandem Solar Cells: The Impact of the Optical Properties and Thickness of the Spacer Layer between Sub-Cells

**DOI:** 10.3390/ma11122570

**Published:** 2018-12-17

**Authors:** Ali Hajjiah, Fahad Parmouneh, Afshin Hadipour, Manoj Jaysankar, Tom Aernouts

**Affiliations:** 1 Electrical Engineering Department, College of Engineering and Petroleum, Kuwait University, Safat 13113, Kuwait; F_kandry_is@hotmail.com; 2 Photovoltaics Department, Thin-Film PV Group, Imec, Partner in Solliance and Energyville, Thor Park 8320, 3600 Genk, Belgium; Afshin.Hadipour@imec.be (A.H.); Manoj.Jaysankar@imec.be (M.J.); Tom.Aernouts@imec.be (T.A.)

**Keywords:** perovskite, solar cells, four-terminal tandem, short-circuit current, light management enhancement, quantum efficiency

## Abstract

Mechanical stacking of a thin film perovskite-based solar cell on top of crystalline Si (cSi) solar cell has recently attracted a lot of attention as it is considered a viable route to overcome the limitations of cSi single junction power conversion efficiency. Effective light management is however crucial to minimize reflection or parasitic absorption losses in either the top cell or in the light in-coupling of the transmitted light to the bottom sub-cell. The study here is focused on calculating an optimum performance of a four-terminal mechanically stacked tandem structure by varying the optical property and thickness of the spacer between top and bottom sub-cells. The impact of the nature of the spacer material, with its refractive index and absorption coefficient, as well as the thickness of that layer is used as variables in the optical simulation. The optical simulation is done by using the transfer matrix-method (TMM) on a stack of a semi-transparent perovskite solar cell (top cell) mounted on top of a cSi interdigitated back contact (IBC) solar cell (bottom cell). Two types of perovskite absorber material are considered, with very similar optical properties. The total internal and external short circuit current (J_sc_) losses for the semitransparent perovskite top cell as a function of the different optical spacers (material and thickness) are calculated. While selecting the optical spacer materials, J_sc_ for both silicon (bottom cell) and perovskite (top cell) were considered with the aim to optimize the stack for maximum overall short circuit current. From these simulations, it was found that this optimum in our four-terminal tandem occurred at a thickness of the optical spacer of 160 nm for a material with refractive index *n* = 1.25. At this optimum, with a combination of selected semi-transparent perovskite top cell, the simulated maximum overall short circuit current (J_sc-combined, max_) equals to 34.31 mA/cm^2^. As a result, the four-terminal perovskite/cSi multi-junction solar cell exhibits a power conversion efficiency (PCE) of 25.26%, as the sum of the perovskite top cell PCE = 16.50% and the bottom IBC cSi cell PCE = 8.75%. This accounts for an improvement of more than 2% absolute when compared to the stand-alone IBC cSi solar cell with 23.2% efficiency.

## 1. Introduction

In the last several years, the photovoltaic industry based on silicon (Si), cadmium telluride (CdTe), and copper indium gallium diselenide (CIGS) has grown rapidly with power conversion efficiency between 14–21% at a cost less than $1/W [1,2]. However, the industry still produces less than one percent of the world electricity with the desire to boost the power conversion efficiency to above 25% and reduce the cost to below $0.5/W [3]. Recently, perovskite has emerged as a promising absorber material for thin-film solar cell technology [4,5]. Solar cells that are based on perovskite have reached a certified 22.1% power conversion efficiency (PCE) in 2016 [6,7]. The perovskite material is based on a small organic cation (A), a cationic group 14 metal (B), and a halide anion (X) to form the ABX_3_ crystal structure. Methylammonium-lead(II)-triiodide with the chemical formula CH_3_NH_3_PbI_3_ (MAPbI_3_) is the most commonly used perovskite semiconductor material in solar cells due to its excellent material properties for applications in photovoltaic. This material is compatible with not only solution processing [8], but also evaporation techniques [9,10]. This direct bandgap semiconductor material [11] has a bandgap of 1.55 eV, which is only ~0.5 V less than its bandgap potential (E_g_/q) [12] and it is tunable to 2.25 eV by substituting Br for I to make MAPb(I_1−x_Br_x_)_3_ [13]. Moreover, this intrinsic material has high carrier mobilities [14], high absorption coefficient [15,16], shallow defect levels [17], and a long charge-carrier diffusion length [18,19,20]. These properties are important for highly performing solar cells and especially attractive for tandem applications. Though, the use of other cations like Cs and formamidinium (FA) instead of methylammonium have led to increased stability and even higher PCE, while maintaining optical properties and bandgap tuning potential [21]. In this study, both MAPbI_3_ as well as CsFAPbIBr will be considered as perovskite absorber material.

One way to increase the efficiency of crystalline Si (cSi) cells is to make tandem solar cells in which a top cell with a higher bandgap than silicon absorbs the higher energy photons. Calculations show that tandem device architecture employing cSi in combination with a wide-bandgap absorber can potentially achieve PCE above 30% [22,23,24,25]. This is due to the reduction in the thermalization loss of charge carriers generated from the high energy photons by the wide bandgap absorber top cell [16,26]. In general, there are two fabrication configurations in tandem solar cells: two-terminal and four-terminal configurations. In the two-terminal configuration, the top solar cell is processed directly on the bottom solar cell, and therefore, the top and bottom cells are electrically connected in series [27,28,29,30]. Hence, the current within both top and bottom cells must be equal, a constraint known as current matching [31]. This strict requirement of current matching constrains the bandgaps of both top and bottom cells, limits the design flexibility, and makes efficiency improvement more difficult. When compared with the two-terminal structure, the four-terminal mechanically-stacked tandem configuration has several advantages. The four-terminal mechanically stacked architecture relaxes the performance constraints, such as current-density matching and the need for tunnel junctions while enabling the optimization of the top and bottom cells separately [32,33,34,35]. In this configuration, the top and bottom cells are electrically isolated from each other, and therefore the output power is extracted separately and enables independent optimization of the solar cell fabrication. Current matching between the top and bottom strings of cells can be achieved at the module level by adjusting the relative top and bottom cell sizes. However, to fully benefit from the four-terminal perovskite/Si tandem cell configuration, choosing the optimum optical spacer and minimizing the parasitic absorption of both the front and rear transparent conductive oxides is crucial for the viability of this tandem configuration [31].

The purpose of the work is to identify the key optical losses in both the optical spacer and the front/rear transparent conductive oxide and optimize the four-terminal perovskite/silicon tandem solar cell structure for maximum overall short circuit current (J_sc-combined, max_) using detailed optical loss analysis.

## 2. Device Architecture and Simulation Procedure

**Figure 1:**
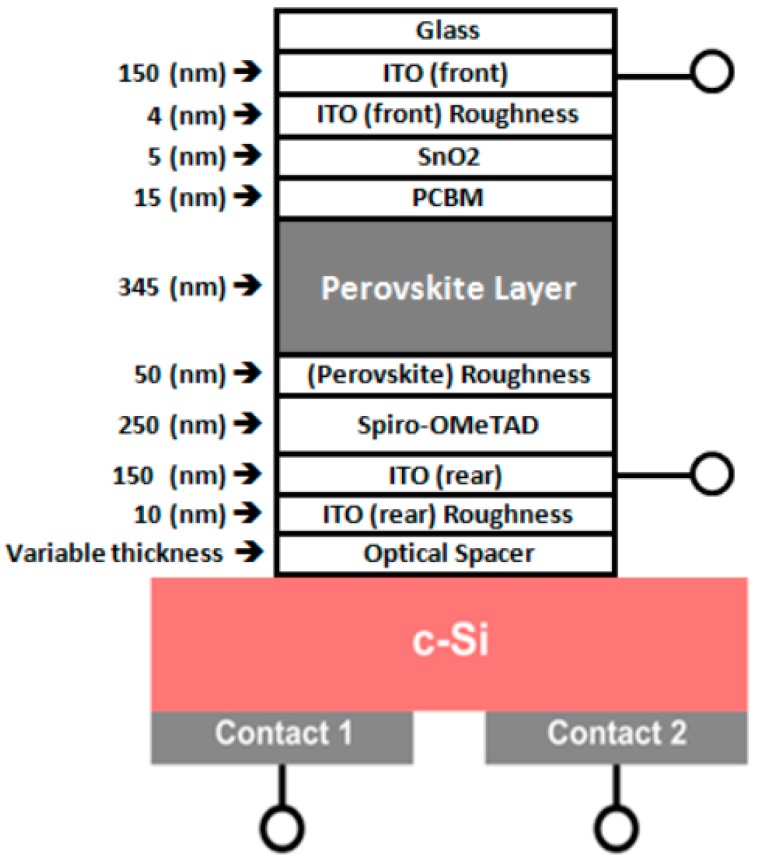
Device structures of the four-terminal perovskite/silicon tandem solar cell are shown.

As mentioned before, two types of perovskite absorber material are used in this study, but incorporated in a similar overall four-terminal perovskite/silicon tandem solar cell structure [36]. In both cases, single junction interdigitated back contact crystalline Si (IBC cSi) solar cell is used as bottom cell. The detailed fabrication process of such cells, prepared from n-CZ (ρ = 1–4 Ω cm) wafers, is described in O’Sullivan et al. [37]. The semi-transparent perovskite top cell has a so-called n-i-p architecture and it consists of 150 nm thick indium–tin oxide (ITO)-coated glass substrate as front electrode, 5 nm thick SnO_2_ combined with 15 nm thick Phenyl-C61-butyric acid methyl ester (PCBM) as electron transport layer, 345 nm thick either MAPbI_3_ or CsFAPbIBr perovskite material as active layers and 250 nm thick 2,2′,7,7′-tetrakis(*N*,*N*-di-p-methoxyphenyl-amine) 9,9′spirobifluorene (Spiro-OMeTAD) as hole transport layer. Finally, 150 nm ITO as rear side transparent electrode completes the planar perovskite solar cell. To conduct optical loss analysis on our four-terminal perovskite/Si solar cell, the Transfer-Matrix-based optical simulation Method (TMM) was used. This method allows for modeling of the optical properties of thin-film layer stacks by solving Maxwell’s equations at each interface through using the complex refractive index and layer thicknesses of all relevant materials as input [38,39,40,41]. More information on the calibration of our (TMM) optical simulation can be seen in [42]. Surface roughness is considered as an effective medium according to the Bruggeman effective medium approximation (BEMA) [43]. Therefore, in our simulations, interface roughness is simulated using a BEMA layer consisting of a mixture of the optical constants for the adjacent media. Schematic model of the four-terminal perovskite/silicon tandem solar cell used in our transfer-matrix-based optical simulations can be seen in Figure 1. The optical coupling of the top perovskite and bottom cSi cell is arranged by an optical spacer material. The four-terminal perovskite/silicon tandem solar cell structure, as depicted here, will be optimized in the optical simulations for maximum overall short circuit current J_sc-combined, max_ by varying the thickness and material of the optical spacer. For this optimization process, both internal and external losses of the top and bottom cell, and the average transmittance of the perovskite top cell in the 800–1200 nm wavelength range are calculated for different materials (different n values) and thicknesses of optical spacer to achieve the maximum overall short circuit current. In general, in a tandem solar cell, the optical interference between transmitted light through one sub-cell with reflected light by another sub-cell will lead to optical cavities [44,45,46]. Due to the creation of optical cavities, the light intensity inside the active layer of the sub-cells can be changed. By using an optimum optical spacer (suitable material with correct thickness), the light intensity that is absorbed by the sub-cells can be maximized, leading, as a result, to maximum performance of the tandem device too, since the efficiency of the tandem solar cell here is the sum of efficiencies of the two sub cells.

The internal and external current losses are calculated using Equations (1)–(3). By integrating the area between the external quantum efficiency (EQE) and the absorbance curves (A_cell_) over the AM1.5G solar spectrum for different wavelength regions, the internal current losses are calculated. For the external current loss calculations, however, losses in both long wavelength light by transmission through the semitransparent ITO-rear to the IBC-Si bottom solar cell through the optical spacer material (J_escape-back_) and losses of light due to external reflection at the device front (J_reflection_) were considered. Subsequently, by using TMM method and Equation (4), J_sc_ (top cell) and J_sc_ (bottom cell) were found, respectively. The sum (J_sc-combined_) of the short circuit currents for both the perovskite (top cell) and the IBC-Si (bottom cell) are calculated and the optimum optical spacer was chosen according to the maximum overall short circuit current.

(1) $$J_{\mathrm{internal-loss}}=\frac{q}{hc}\int_{\lambda_{1}}^{\lambda_{2}}\lambda \times \Phi \left(\lambda \right)\times \left[A_{\mathrm{top}\;\mathrm{cell}}\left(\lambda \right)-\mathrm{EQE}\left(\lambda \right)\right]\times d\lambda$$

(2) $$J_{\mathrm{escape-back}\;\mathrm{external}}=\frac{q}{hc}\int_{500}^{1000}\lambda \times \Phi \left(\lambda \right)\times T_{\mathrm{top}\;\mathrm{cell}}\left(\lambda \right)\times d\lambda$$

(3) $$J_{\mathrm{reflection}}=\frac{q}{hc}\int_{\lambda 1}^{\lambda 2}\lambda \times \Phi \left(\lambda \right)\times R\left(\lambda \right)\times d\lambda$$

(4) $$J_{\mathrm{sc-Si}\left(\mathrm{bottom}\right)}=\frac{q}{hc}\int_{300}^{1200}\lambda \times \Phi \left(\lambda \right)\times T_{\mathrm{top}\;\mathrm{cell}}\left(\lambda \right)\times EQE_{\mathrm{bottom}\;\mathrm{cell}}\left(\lambda \right)\times d\lambda$$

where q is the elementary charge, h is Planck’s constant, c is the speed of the light, λ is the wavelength, Φ(λ) is the AM1.5G solar spectrum, T_cell_ is the transmission of the cell, and R is the reflection of the cell.

## 3. Results and Discussions

**Figure 2:**
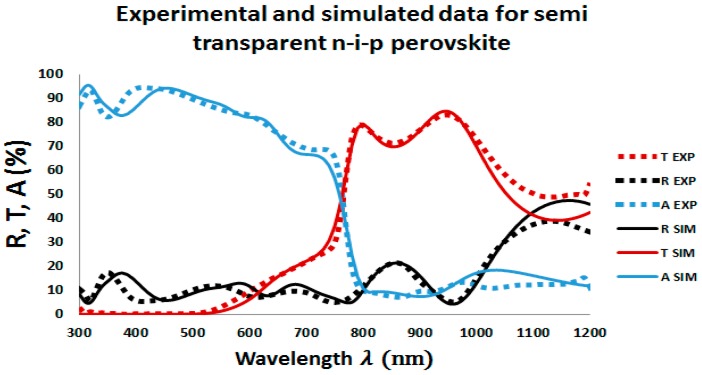
Measured and simulated reflectance (R), transmittance (T), and absorptance (A) spectra of semitransparent MAPbI3 solar cell with the architecture glass/ITO-front/ITO-front-roughness/SnO2/PCBM/MAPbI3/MAPbI3-roughness/spiro-OMeTAD/ITO-rear/ITO-rear-roughness. The dashed lines represent measurements on the actual device and solid lines represent transfer-matrix-based simulations.

First, in order to calibrate and underline the accuracy of our optical simulations, Reflectance (R), Transmittance (T), and Absorptance (A) of a semi-transparent perovskite-based sub cell with MAPbI_3_ as absorber material in an architecture of the perovskite cell, as depicted in Figure 1, were measured and compared with simulations. Excellent agreement with experimental data can be seen in Figure 2, which confirms the accuracy of our simulation. However, the small offsets for long wavelengths in our transmission and reflection plots between experimental and simulation data are due to the small absorption or scattering effects in the substrate.

**Figure 3:**
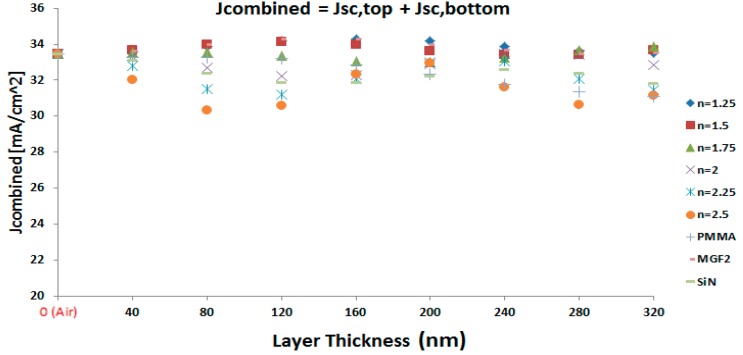
The sum of short-circuit current of sub-cells of tandem device is shown for optical spacers with different thickness and different refractive index’s (*n*).

With the accuracy of the optical simulation method validated, the full stack of the four-terminal perovskite/cSi tandem is considered with MAPbI_3_ as an active layer for the perovskite sub-cell, the internal and external losses are calculated such that the short circuit currents for both the top and bottom cell can be determined. Thereby, the optical properties (*n* value) and thickness of the spacer layer is varied to find the overall combined short circuit current as the sum of the photo-currents generated in both sub-cells. In our optical simulations, the refractive index n of the spacer layer is varied over a broad range from *n* = 1.25 to *n* = 2.5, since simulation gives us the freedom to choose any arbitrary value for n. In order to address the real working device, as examples, the optical data from common optical spacer materials, like Silicon Nitride (SiN; *n*(SiN) = 2.0458), Magnesium Floride (MgF_2_; *n*(MgF_2_) = 1.3777), and polymethylmetacrylate (PMMA; *n*(PMMA) = 1.4906) are used as well in the simulations. In general, optical mediums, such as SiN and MgF_2_, can be coated by sputtering [47,48], thermal evaporation [49,50,51], and atomic layer deposition (ALD) [52] while polymer-based spacers such as Polymethylmethacrylate (PMAA) can be coated by solutions using different methods, such as spin, blade, slot die, and spray coating [53]. The spacer thickness is varied from 0 nm up to 320 nm, in steps of 40 nm. The resulting overall combined short circuit current is represented in Figure 3 for each of these combinations of n value and layer thickness.

It can be observed that the variation of the *n* value can have a substantial effect, resulting in a variation of the overall combined short circuit current of almost 5 mA/cm². With a layer thickness of the optical spacer of 80 nm, the J_sc-combined_ is as low as 30 mA/cm², while it rises over 34 mA/cm² for *n* = 1.5 at the same spacer layer thickness. Furthermore, it can be observed that this variation is large for either relatively low spacer thicknesses of 80 or 120 nm, or for higher thickness values, like 280 and 320 nm. The variation is lowest for 200 nm spacer layer thickness. Another observation is that for thickness below 160 nm, J_sc-combined_ is the largest for *n* = 1.5 each time, while from that thickness up to 240 nm *n* = 1.25 results in the largest J_sc-combined_.

**Figure 4:**
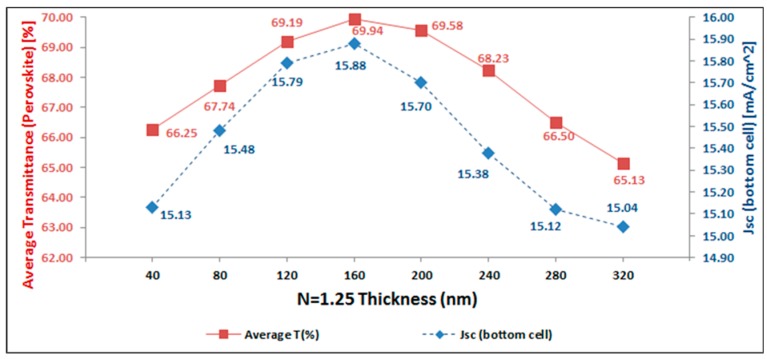
Optical transmission of top sub-cell based on semi-transparent perovskite solar cells (red) and short-circuit current of interdigitated back contact crystalline Si (IBC c-Si) bottom sub-cell are shown.

Finally, as it is clear from Figure 3, the total photo-current will be maximum for the simulated four-terminal tandem device, when an optical spacer is used with *n* = 1.25 and a thickness of 160 nm. Further analysis of the simulation data, as depicted in Figure 4, clarifies that at these conditions the average optical transmittance of the perovskite-based sub-cell is reaching a maximum, just below 70%. This in turn results in a maximum value for the short-circuit current of the IBC cSi bottom sub-cell, close to 16 mA/cm². This is an excellent illustration of the importance of the light management by the optical spacer layer to not only maximize the current generation in the top cell, but also in maximizing the light in coupling into the bottom cell to simultaneously have high current generation in that sub-cell.

**Figure 5:**
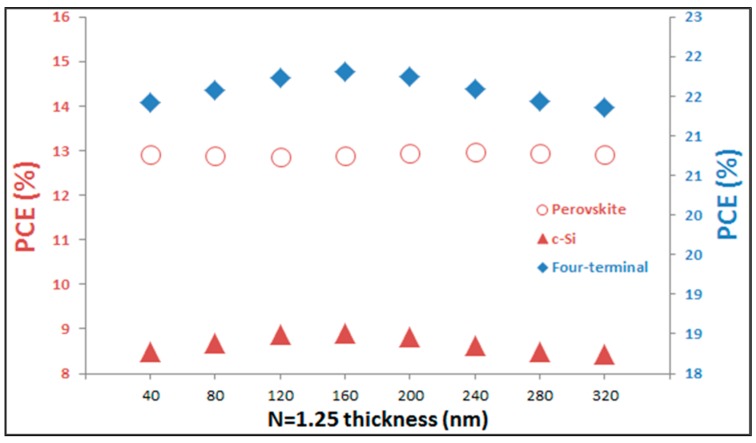
The performance of sub-cells is compared with four-terminal tandem device for optical spacer with refractive index of *n* = 1.25 but different thicknesses.

This observation is further validated in Figure 5, where the PCE values of both sub-cells as well as the full four-terminal tandem are calculated for different thickness of the optical spacer layer, with *n* = 1.25. We assume open-circuit voltage (V_OC_) equal to 0.97 Volt and Fill Factor (FF) equal to 72% as taken from reference [36] as an example (when MAPbI_3_ is used as active layer of perovskite based sub-cell) and use our simulated short-circuit currents (J_SC_) to calculate the efficiencies of sub-cell and compare them with the four-terminal tandem device. The data clearly indicate that, while there is minor variation in PCE for the perovskite based sub-cell, the variation for the cSi sub-cell follows the J_sc_ trend, as shown in Figure 4. It is this variation of J_sc_ that subsequently results in the maximum overall PCE value of the four-terminal tandem device, close to 22%.

**Figure 6:**
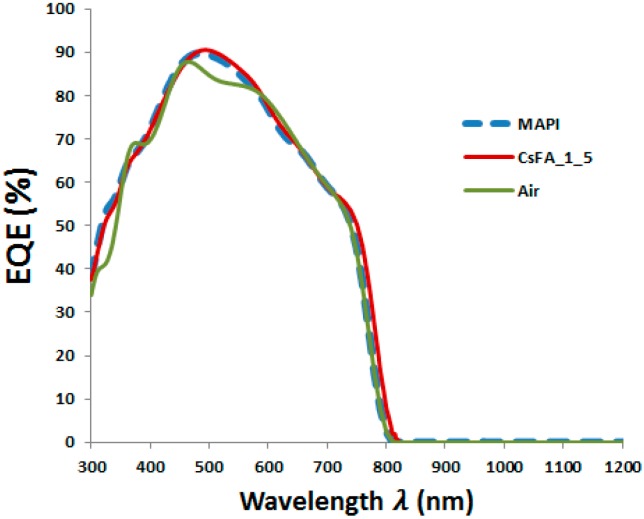
Simulated external quantum efficiency (EQE) solar cells based on MAPbI_3_ and CsFAPbIBr perovskites.

As the IBC cSi single cell shows an efficiency of 23.2%, it means that the four-terminal tandem device, as simulated here, is not yet outperforming this cSi single cell. To further increase the performance of the resulting tandem structure, we investigate the effect of replacing the perovskite absorber layer from MAPbI_3_ to an electrically better performing one, such as CsFAPbIBr type [54]. MAPbI_3_ and the CsFAPbIBr type selected have almost identical bandgaps equal to 1.55 eV and 1.56 eV, respectively, and hence have very similar optical performance. Based on purely optical simulations, the simulated EQE of MAPbI_3_ and CsFAPbIBr solar cells are almost identical, as depicted in Figure 6, suggesting that both devices have almost identical J_SC_. However, CsFAPbIBr solar cells show higher V_OC_ (1.12 V) and fill factor (78%), thus resulting in higher PCE than MAPbI_3_ solar cells with similar device structure [54].

By replacing MAPbI_3_ with the CsFAPbIBr perovskite [55,56], we simulate the four-terminal tandem stack again, with the optical spacer layer thickness at 160nm and its refractive index *n* = 1.25, as determined before. With similar J_sc_ for the top cell but higher V_oc_ and FF, the PCE for the CsFAPbIBr based perovskite sub-cell is almost 4% higher than the MAPbI_3_ based one, up to 16.5%, from a previous 12.9%. With the optical transmittance similar as before, the cSi bottom sub-cell maintains a PCE of 8.75%, only marginally lower than the 8.91% value when using the MAPbI_3_ sub-cell. Replacing MAPbI_3_ with the CsFAPbIBr perovskite therefore results in an overall four-terminal tandem performance as high as 25.26%, performing substantially better than the IBC cSi stand-alone cell. These values are summarized in Table 1.

**Figure 7:**
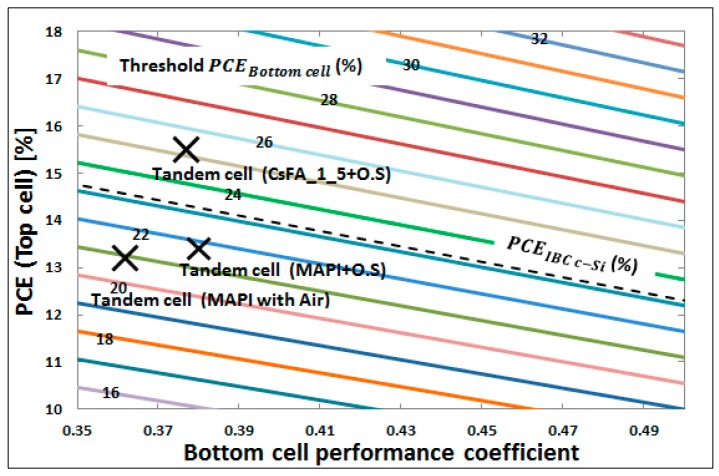
Performances of sub-cells are compared with four-terminal tandem device based on two type of perovskite based solar cells and the same IBC cSi solar cell. Note: O.S. in the figure stands for optimized optical spacer.

Thus, this study clarifies two main pathways to identify four-terminal perovskite/cSi tandem devices that can outperform the stand-alone cSi cell, as depicted in Figure 7. Here, in order to determine the factor by which the Si cell PCE drops when in tandem configuration when compared to stand-alone, the bottom cell performance coefficient (γ) is calculated. This coefficient is influenced by the sub-bandgap transparency of the top solar cell, and it is calculated as the ratio of the PCE of the bottom solar cell in four-terminal configuration to its stand-alone PCE, see Equation (5).

(5) $$\gamma =\frac{{\mathrm{PCE}}_{\mathrm{bottom}\_\mathrm{cell}\left(\tan\mathrm{dem}\right)}}{{\mathrm{PCE}}_{\mathrm{stand}\_\mathrm{alone}}}$$

The colored diagonal lines indicate how the four-terminal tandem PCE varies as function of this coefficient and the top cell PCE. We have demonstrated with the simulations done at first with the MAPbI_3_ based perovskite top cell, this bottom cell performance coefficient can be substantially improved when optimizing the light in-coupling with an optical spacer layer with appropriate refractive index value and thickness. For an almost identical top cell PCE of ~13.5%, the coefficient shifts from ~0.36 to almost 0.39 with the optimized optical spacer, as indicated by the two MAPI-labeled crosses. To outperform the IBC cSi stand-alone PCE, it turns out to be crucial to maintain the bottom cell performance coefficient while increasing the top cell PCE, here to a value of ~16% (CsFA labeled cross), as we achieved by replacing MAPbI_3_ with the almost optically similar CsFAPbIBr perovskite.

## 4. Conclusions

Through the use of the transfer matrix optical simulation method, we have demonstrated the impact of the optical properties and thickness of the spacer layer in between the top and bottom sub-cells on the overall performance of a four-terminal perovskite/cSi tandem device. At first, it is calculated that the optical spacer layer with appropriate refractive index and thickness enhances the light transmission from the top to bottom cell, and thereby substantially improves the bottom cell performance coefficient. In a second step, the top cell absorber was replaced with an optically similar but electrically higher performing perovskite material. Maintaining the optical light in-coupling, this resulted in a four-terminal tandem structure that outperforms the IBC cSi stand-alone cell with more than 2% absolute. Therefore, this illustrates that the optical simulation method described here can contribute well for optimizing the whole tandem structure reducing time and cost of optimizing methods based on pure experiments. Moreover, further simulation work will be targeted to optimize the bandgap of the perovskite top cell to further improve the bottom cell performance coefficient.

## Figures and Tables

**Table 1:** The efficiencies of single sub-cells and four-terminal tandem device are presented based on experimental values from literature (reference [36] and reference [54]) for V_oc_ and Fill Factor (FF), but simulated results for J_sc_ (this study).

Perovskite	PCE %			
	Perovskite Top-Cell	IBC cSi Bottom-Cell	Four-Terminal with Perovskite Top- and IBC cSi Bottom-Cell	IBC cSi (Alone)
MAPI	12.90	8.91	21.81	23.2
CsFA_1_5	16.50	8.75	25.26	23.2
